# Emotional Experience, Physiological Reactivity, and Psychological Difficulties during middle childhood: a pilot study

**DOI:** 10.1192/j.eurpsy.2023.1535

**Published:** 2023-07-19

**Authors:** S. Charpentier-Mora, M. Tironi, F. Bizzi

**Affiliations:** University of Genova, Genova

## Abstract

**Introduction:**

Many studies linked expression and regulation of emotions as transdiagnostic variables involved in the emergence of psychopathological disorders. However, few studies investigated such processes during middle childhood, a critical period of life defined by significant psychological, physical, and social changes.

**Objectives:**

Explore the relationship between child’s emotion regulation – both emotional subjective experience and physiological reactivity – and child’s psychological difficulties.

**Methods:**

20 children (M_age_=10.7, SD=1.25; 65% males) and their mothers were recruited from general population. Emotion regulation was assessed with (1) *How I Feel* (HIF) and *Positive and Negative Affect* questionnaires (PANAS) and (2) Heart Rate (i.e., Beats Per Minute, BPM) and Heart Rate Variability (i.e., High Frequency and Low Frequency ratio, LF/HF). Child’s psychological difficulties were measured with the parent report *Child Behavioral Checklist 6-18* (CBCL-6/18).

**Results:**

Statistically significant correlations emerged between the HIF-Positive emotions scale and both externalizing (*rs* = -.51) and internalizing (*rs* = -.46) difficulties; the HIF-Negative emotions scale and internalizing difficulties (*rs* = .49); LF/HF and internalizing difficulties (rs = -.58). Finally, a non-significant but moderate effect was found between the HIF-Negative emotions scale and externalizing difficulties (*rs* = .33).

**Conclusions:**

Although the limited number of participants, data suggest an interesting role played by both child’s emotional experience and physiological reactivity on internalizing and externalizing difficulties as reported by mothers. More specifically, child’s experience of positive emotions is associated with fewer internalizing and externalizing difficulties, while child’s experience of negative emotions illustrates an opposite relationship, implying the relevance of looking at child’s emotional subjective experience in understanding psychological difficulties. Moreover, LF/HF ratio – labeled as the child’s sympathovagal balance – seems like it might be higher in children with less internalizing difficulties. Although doubts about LF/HF interpretation, several studies share this view showing a decrease in autonomic reactivity in internalizing problems, such as depression, in adults. Overall, our preliminary results underline the importance of studying the emergence of psychopathological outcomes in middle childhood connected to both psychological and physiological emotional processes.

**Disclosure of Interest:**

None Declared

